# Correlation Dimension Detects Causal Links in Coupled Dynamical Systems

**DOI:** 10.3390/e21090818

**Published:** 2019-08-21

**Authors:** Anna Krakovská

**Affiliations:** Institute of Measurement Science, Slovak Academy of Sciences, 841 04 Bratislava, Slovakia; krakovska@savba.sk

**Keywords:** causality, correlation dimension, common driver, the arrow of time

## Abstract

It is becoming increasingly clear that causal analysis of dynamical systems requires different approaches than, for example, causal analysis of interconnected autoregressive processes. In this study, a correlation dimension estimated in reconstructed state spaces is used to detect causality. If deterministic dynamics plays a dominant role in data then the method based on the correlation dimension can serve as a fast and reliable way to reveal causal relationships between and within the systems. This study demonstrates that the method, unlike most other causal approaches, detects causality well, even for very weak links. It can also identify cases of uncoupled systems that are causally affected by a hidden common driver.

## 1. Introduction

Causality, as a relation between cause and effect, is a complex topic discussed from many perspectives, starting from situations of everyday life, through philosophy, to mathematics and physics.

In the last few decades, new causal methods are continually being designed and at the same time, there is an ongoing debate over whether or not the relationships found using the individual methods are actually causal. The interested reader can learn more from the book of Pearl and Mackenzie [1]. As the authors emphasize in the book, several operational methods exist for discovering potential causal relations. However, regardless of language, reasoning through algorithms of inductive causation should follow to determine which connections can be inferred from empirical observations even in the presence of latent variables. The concept reveals the limits of what can be learned by causal analysis from observational studies and represents a more general strategy comprising approaches used so far. However, the development of successful techniques, such as times series causality tools, remains useful as these tools help to draw potential causal links that are later verified by means such as Pearl’s model.

In this study, we will present one of such tools, that is applicable in a special case—when we ask about the causal relationship between dynamical systems.

We restrict ourselves to the study of causality in situations where processes are represented by time series. Following the concept, introduced by Clive Granger in 1969, we respect that an effect cannot occur before its cause and we say that *x* causes *y* if, information on the recent past of the time series *x* helps improve the prediction of *y* [2]. Comparison of several causal methods in [3] has shown that the Granger causality test works well for autoregressive models but produces false positives for time series coming from coupled dynamical systems. Some more recent information-theoretic methods such as conditional mutual information or transfer entropy [4,5] are usually more successful with data from dynamical systems. Methods that work in reconstructed state spaces may also be used successfully. This includes the method of predictability improvement, which determines whether a prediction of an observable from system *Y*, made in a reconstructed state space, improves when observable from system *X* is included in the reconstruction. If the predictability improves, then, analogously to the idea of Granger’s causality in the case of autoregressive processes, we hypothesize that *X* causes *Y* [3].

However, Cummins et al. have introduced a comprehensive theory showing that in coupled dynamical systems we have limited possibilities when it comes to uncovering causal links. The best we can hope for is finding the strongly connected components of the graph (sets of mutually reachable vertices) which represent distinct subsystems coupled through one-way driving links [6]. We cannot identify self-loops, and, we cannot distinguish the direct driving, indirect driving, correlate of direct driving and correlate of an indirect driving.

In this study, we would like to draw attention to an interesting new way to reveal detectable causal relationships in dynamical systems. The method makes use of the fact that the driver has lower complexity and degree of freedom than the driven system containing information about the driving dynamics. To quantify the complexity, the so-called correlation dimension ($D_{2}$) will be used.

The use of the correlation dimension in the context of causality detection has been proposed by Janjarasjitt and Loparo in 2008 [7]. The study has built on the fact that, if the subsystems *X* and *Y* are independent, then the active degrees of freedom of the combined system is equal to the sum of the active degrees of the subsystems. On the other hand, if *X* and *Y* are coupled, the active degrees of freedom of the combination is expected to be reduced. The authors have introduced an index named dynamic complexity coherence measure. It has been defined as the ratio of the sum of the correlation dimensions of the individual subsystems to the correlation dimension of the combination of the subsystems. It has been shown that the index is useful to quantify the degree of coupling because it is increasing with intensifying coupling strength. The index was designed to detect the presence of coupling but not to determine the direction of the link. Regarding the direction, the authors have suggested utilizing the variability in the correlation dimension, which is supposed to be greater in the case of the response system than in the driving system alone.

However, we have shown in 2013 that not just the presence but also the direction of the link is simply identifiable from the $D_{2}$ estimates [8]. The study has demonstrated that $D_{2}$ for a unidirectionally driven system and the combined state portrait are equal and significantly higher than $D_{2}$ for the driving system. Bidirectional causality and synchronized dynamics, on the other hand, are characterized by the equality of all three evaluated dimensions.

Finally, in a recent study [9], the authors pointed out that even the hidden common driver of the systems *X* and *Y* can be detected through estimates of fractal dimensions (they used the information dimension), although it must always be emphasized that this is only possible in cases where *X* and *Y* are not interconnected.

In this study, the topic of using the fractal dimension for causality detection is revisited. Our goal is to systematically specify detectable types of causal relations, test the $D_{2}$-based method for different levels of coupling strength, and highlight the potential of the methodology.

In the following section, we focus on the choice of the so-called embedding parameters for the reconstruction of the examined dynamics, as the quality of the reconstruction is essential for the accuracy of dimension estimation. Then the $D_{2}$-based method of causality detection is explained. Finally, test examples of different causal relationships are given and the results are presented. The sensitivity of the method to the strength of the links is also discussed. We also briefly comment on the $D_{2}$-based approach concerning the current topic of causal analysis of time-reversed measurements.

## 2. Methods

### 2.1. State Space Reconstruction

The goal of this study is to use $D_{2}$ to identify the causal relationship between dynamical systems *X* and *Y*, represented by time series *x* and *y*, respectively. Since we are interested in the correlation dimension for dynamics of the whole systems, as a first step, an $m_{X}$-dimensional manifold $M_{X}$ is reconstructed from lags of observable *x* so that the state of the system in time *t* is $[x\left(t\right),x\left(t-\tau_{X}\right),x\left(t-2\tau_{X}\right),\ldots ,x\left(t-\left(m_{X}-1\right)\tau_{X}\right)].$ Using $\tau_{Y}$ and $m_{Y}$, the manifold $M_{Y}$ is reconstructed analogously. Given certain conditions, the reconstructed manifold is, in the sense of diffeomorphism, equivalent to the original one [10]. Consequently, they share the same features in many ways. Most importantly for us, the reconstruction preserves relevant geometrical and dynamical invariants, including the fractal dimension.

Theoretically, for noise-free data of unlimited length, the existence of a diffeomorphism between the original attractor and the reconstructed image is guaranteed for a sufficiently high embedding dimension *m* and almost any choice of delay τ. The theorem of Whitney (called Embedding theorem in [11]) guarantees the possibility of embedding any *d*-dimensional smooth manifold into m=(2d+1)-dimensional Euclidean space. Sauer et al. have generalized this theorem to fractal objects [12]. The authors have proved that, under some conditions regarding periodic orbits and the measurement function, almost every $C^{1}$ map from the fractal *A* to $R^{m}$ with $m>2D_{A}$ forms an embedding, whereby $D_{A}$ is the box-counting dimension of *A*. This finding means that it is not the size *d* of the manifold of the original attractor that determines the minimal embedding dimension but only the fractal dimension $D_{A}$. However, even $2D_{A}$ represents only an upper limit—the embedding theorem does not rule out an embedding dimension that is, in some situations, lower than $2D_{A}$ (occasionally as low as the dimension of the original system).

Sometimes, the sufficient size of the reconstruction space can be smaller than $2D_{A}$ because of the less demanding goal of the investigation. For example, for the numerical estimation of the correlation dimension of the attractor *A*, any dimension above the box-counting dimension of *A* is enough [13]. Of course, such cases do not guarantee that the original attractor is mapped one-to-one onto its reconstructed version; however, that is not necessary for dimension estimation.

In real-world applications, the time series can be short and noisy. Then, the quality of reconstruction varies greatly depending on the value of τ and *m* and it is worthwhile to pay close attention to the selection of these parameters. The simple idea is to unfold the reconstruction of the trajectories sufficiently to avoid self-crossings and extreme closeness of distinct parts. To achieve this, the first minimum of the mutual information is usually used to estimate τ and the false nearest neighbor test to find the sufficient *m* [14]. However, it should be emphasized that using mutual information for the selection of the time delay can be regarded as effective only for a time series that has a single, dominant periodicity or recurrence time. In this case, the suitable lag is approximately one-quarter of the dominant period, and this value is also in good agreement with the minimum of the mutual information or the first zero of the auto-correlation function. This is true for a two-dimensional plot. However, the same delay time is often used regardless of the number of delay vectors that form the reconstruction, although some authors suggest lowering the delay time when increasing the embedding dimension. They argue that the independent parameter that should be estimated is not the delay τ or the embedding dimension *m* separately but rather the whole embedding time window mτ. As demonstrated in [15], the appropriate time window seems to be between the quarter and half of the mean orbital period, if the period can be approximated by examining the oscillatory patterns in the data.

However, data can be broadband and lacking any indication of periodicity. Then, what we can do, is to follow some proper invariant that is expected to reach an extremum for a suitable combination of τ and *m*. For example, the predictability or percentage of false nearest neighbors can be evaluated for several combinations of dimensions and delays to reveal the combination that leads to the best achievable results [15]. In this way, the most appropriate τ and *m* are found in one step. Finally, recall that finding a suitable time window does not mean that much larger than necessary *m* (with an appropriately reduced τ) can be used equally well for the analysis of the underlying dynamics. With a limited amount of data, the points become sparse in too large embedding space, which spoils the results. Consequently, using the selected embedding window, but with a preference for lower dimensions, leads to the best results.

After finding a suitable embedding window and reconstructing the state portraits, we can move on to the $D_{2}$ estimating and then proceed with the detection of causal links.

### 2.2. Correlation Dimension Estimation

Fractal dimension is an important geometrical quantity characterizing the degree of complexity and possible fractal nature of the attractor of the studied dynamical process. There are many ways to define and estimate the fractal dimension. However, since 1983, the most commonly used one is the computationally efficient approach of Grassberger and Procaccia [16]. The method involves estimating the correlation sum

$$C\left(r\right)=\frac{2}{N(N-1)}\sum_{i=1}^{N}\sum_{j=i+1}^{N}\Theta \left(r-\left|\right|x_{i}-x_{j}\left|\right|\right),$$

where *N* is the number of points on the attractor, and Θ is the Heaviside function (1 for non-negative argument, 0 for negative argument). C(r) can be understood as the probability that the distance of two points $x_{i}$, $x_{j}$, randomly selected on the attractor, is less than *r*.

One then inspects the scaling relation

$$C\left(r\right)\propto r^{D_{2}}\;\mathrm{for}\;r\to 0,$$

leading to correlation dimension

$$D_{2}=\lim_{r\to 0}\frac{\partial \ln(C(r))}{\partial \ln(r)}.$$

In practice, this means that to find the correlation dimension, we have to plot lnC(r) as a function of lnr and follow the slope of the obtained curve. This slope is called correlation exponent, and its limit for decreasing *r* corresponds to the correlation dimension.

However, recall that detection of the limit for decreasing *r* is impossible for a small amount of data [17]. Therefore, we will emphasize repeatedly the need for long time series, meaning at least $10^{m}$ data points to estimate $D_{2}$ in *m*-dimensional state space.

### 2.3. Causality Detection Based on Correlation Dimension

As mentioned in the introduction, we have used the correlation dimension in causal analysis already in [8], with inspiration coming from [7]. We have also used $D_{2}$ in 2015 in nine tested examples of coupled chaotic systems to identify the level of coupling strength leading to synchronization [18]. In this subsection, we present a completed method of using $D_{2}$ to detect causal relationships in interconnected dynamical systems.

The method builds on strong theoretical foundations discussed above—on the Takens’ theorem, which allows reconstructing the state portrait of underlying dynamics from a single observable [10], the work of Sauer et al., which tells us more about the fractal dimensions of reconstructed attractors [12], and the work of Cummins et al. setting limits on what can be revealed about causal relationships in dynamical systems [6].

Suppose in the following that we have two dynamical systems *X* and *Y* and each one is represented by one time series, *x*, and *y*, respectively. First of all, we find suitable embedding parameters *m* and τ for each system to make reconstructions of the manifolds $M_{X}$ and $M_{Y}$. The next step is to estimate the correlation dimension of the reconstructed objects. It is obvious that the dimension of the unidirectionally driven dynamical system cannot be lower than the dimension of the driving system. However, comparing these two dimensions would not be enough to draw conclusions about causal relationships. We need to join the reconstructions $M_{X}$ and $M_{Y}$ into $M_{\left[XY\right]}$, where the pair of square brackets is the concatenation operator. Here we concatenate matrices horizontally, which means that they must have the same number of rows and the resulting matrix has $m_{X}+m_{Y}$ columns. The correlation dimension computed for $M_{\left[XY\right]}$ is a good approximation of the active degrees of freedom of the combination of the two systems.

Then, the next causal relations between *X* and *Y* with corresponding options for the correlation dimensions $D_{2}\left(X\right)$, $D_{2}\left(Y\right)$ and $D_{2}\left(\left[XY\right]\right)$ are possible:

#### 2.3.1. X→Y or Y→X

As a first example let us have a unidirectional link from driving system *X* to response *Y*. Then the equations describing the dynamics of the driven *Y* include the equations for the separate *X* and the equations for the interconnection of both systems. Consequently, according to Takens’s theorem, reconstruction of the entire driven system *Y* from any *y*-coordinate includes information about the *X* dynamics and also information about the interconnection of the two systems. Therefore, $D_{2}\left(Y\right)$ cannot be lower than $D_{2}\left(X\right)$ and the correlation dimension estimated from $M_{\left[XY\right]}$ is the same as the estimate from $M_{Y}$: $D_{2}\left(\left[XY\right]\right)=D_{2}\left(Y\right)>D_{2}\left(X\right)$.

In the opposite case of Y→X the $D_{2}$ estimate from $M_{\left[XY\right]}$ equals the estimate from $M_{X}$ and they are higher than the dimension of the driver *Y*.

#### 2.3.2. *X* and *Y* Are Independent

For uncoupled, mutually independent *X* and *Y* the correlation dimension of the combined system is expected to be equal to the sum of the dimensions of *X* and *Y*.

#### 2.3.3. Uncoupled *X* and *Y* with a Hidden Common Driver

In the third option, similarly as in the previous one, the time series *x* and *y* come from systems *X* and *Y* that are mutually independent. In this case, however, both *X* and *Y* are controlled by a common hidden driver *Z* for which we have no information. Some causal methods falsely identify such processes as being causally linked (see e.g., [3]).

We expect the active degrees of freedom of $M_{\left[XY\right]}$ reduced compared to the previous test example. This is because the complexity of the hidden driver *Z* contributes twice to the sum $D_{2}\left(X\right)+D_{2}\left(Y\right)$, but only once to $D_{2}\left(\left[XY\right]\right)$. Consequently, the presence of a hidden common cause without a direct causal effect between the two systems is indicated by the joint dimension less than the sum of the single dimensions but higher than either of them.

Note that the common driver is only detectable in the case of uncoupled processes. If the common cause coexists with a unidirectional or bidirectional link between *X* and *Y*, then the coupling will ensure the transfer of the common driver information and, as a result, the driving links will be detectable, while the common driver remains hidden.

#### 2.3.4. X↔Y

The last option refers to the situation with a cyclic flow of information when *X* and *Y* are bidirectionally linked. In such cases, the causes and effects are entangled and, based on the Takens’ theorem, the whole underlying dynamics is reconstructable from any measured *x* or *y* observable [10]. The reconstructed manifolds are equivalent, having equal dimensions and the same dimension applies to the joint reconstruction, i.e., $D_{2}\left(\left[XY\right]\right)=D_{2}\left(X\right)=D_{2}\left(Y\right)$.

The rules for inferring causal relations from $D_{2}$ are summarized in Table 1.

If, thanks to a large number of data, we can afford the luxury of repeated $D_{2}$ estimates, then non-parametric statistical tests can be used to evaluate the significance of the results. However, even if we have only one estimate of $D_{2}\left(X\right)$, $D_{2}\left(Y\right)$, and $D_{2}\left(\left[XY\right]\right)$, we know that the expected integer gap between the degrees of freedom of cause and effect in unidirectional connection represents a significant difference. This is also reflected in a substantial distance between the dimensions, and therefore occurrences of false conclusions are unlikely. However, only future tests and extensive experience with real data can assess the true reliability and robustness of the method.

## 3. Results

The $D_{2}$-based causality detection applies to any pair of time series that originate from dynamical systems and are long and clean enough to allow reasonably accurate estimates of the correlation dimensions of systems.

To demonstrate the ability of $D_{2}$ to detect causal relations we decided to use selected pairs of time series produced by the well-known chaotic Hénon maps.

First of all, from the time series, we made reconstructions of the state portraits. To do so, we explored a suitable invariant (number of false nearest neighbors or the predictability) for several combinations of parameters *m* and τ and selected the one that led to the best result (minimum of false neighbors or the lowest prediction error) [15].

Then we estimated $D_{2}\left(X\right)$, $D_{2}\left(Y\right)$, and $D_{2}\left(\left[XY\right]\right)$ for the reconstructed state portraits and derived the corresponding causal relation between *X* and *Y*.

This section presents the individual test examples, the outputs obtained for each test case, and the visualization of the results.

### 3.1. X→Y

Equation (1) represents our first test example—unidirectional driving of system *Y* by *X*. The first two lines correspond to the driving Hénon map *X*, and the following two equations describe the response *Y*:

(1) $$\begin{array}{cc} x_{1}\left(t+1\right) & =1.4-x_{1}^{2}\left(t\right)+0.3x_{2}\left(t\right)\\ x_{2}\left(t+1\right) & =x_{1}\left(t\right)\\ y_{1}\left(t+1\right) & =1.4-\left(Cx_{1}\left(t\right)y_{1}\left(t\right)+\left(1-C\right)y_{1}^{2}\left(t\right)\right)+0.3y_{2}\left(t\right)\\ y_{2}\left(t+1\right) & =y_{1}\left(t\right) \end{array}$$

*C* controls the strength of the coupling, with C=0 for uncoupled systems. Plots of conditional Lyapunov exponents in [19], similarly as correlation dimension estimates in [18], show that synchronization takes place at about C=0.7.

**Figure 1:**
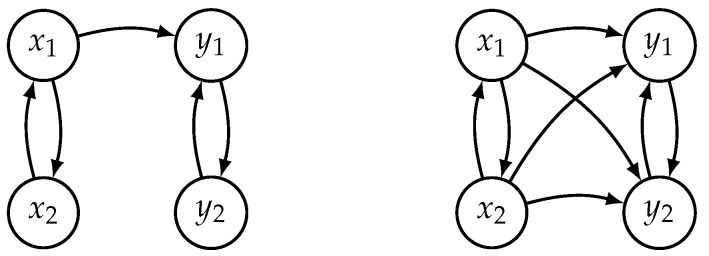
Interaction graph for unidirectional coupling of two Hénon systems described by Equation (1) (on the left) and the connections detected by causal analysis in reconstructed state spaces (on the right).

Before we start with a causal analysis of this example, let us remember some of the limits we are encountering here. The so-called interaction graph (see the left part of Figure 1), which is easy to interpret from Equation (1), shows how *X* and *Y* are coupled through a one-way driving relationship between variables $x_{1}$ and $y_{1}$. In an interaction graph, the nodes representing the variables are connected by directed edges whenever one variable directly drives another.

Now imagine that we have the time evolution of all four variables, but we do not know how they are linked. According to Cummins et al. [6], complete pair-wise causal testing should reveal all five links in the left graph of Figure 1. In addition to these, since we cannot distinguish between direct and indirect driving, we would also see $x_{1}$ driving $y_{2}$ and $x_{2}$ driving both $y_{1}$ and $y_{2}$ (see the right graph of Figure 1). In summary, we would correctly find that $x_{1}$ and $x_{2}$ form one subsystem *X*, $y_{1}$ and $y_{2}$ form the second subsystem *Y*, and X→Y. However, the exact position of the direct driving link ($x_{1}\to y_{1}$) cannot be determined.

The test example given by Equation (1) for increasing coupling strengths has been recently analyzed with six different causal methods [3]. The methods included the Granger VAR test, the extended Granger test, the kernel Granger test, cross-mapping techniques, conditional mutual information, and assessment of the predictability improvement. Detailed results can be found in the extensive supplemental material of the article [3]. The study has shown, among other things, that the Granger test does not apply to data from dynamical systems and even some of the popular methods, supposedly suitable for analysis of dynamical systems, have extremely low specificity—–they produce a large number of false detections of causality.

**Figure 2:**
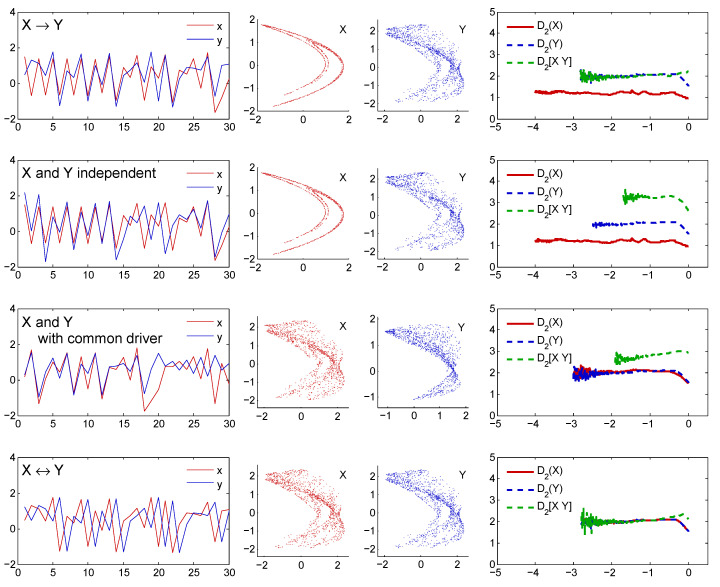
Four examples of detectable types of causal relations between time series. Each row contains plots of 30 points of investigated time series *x* and *y*, two-dimensional projections of the reconstructed state portraits of systems *X* and *Y*, and the plateaus of the correlation exponents used to estimate $D_{2}\left(X\right)$, $D_{2}\left(Y\right)$, and $D_{2}\left(\left[XY\right]\right)$.

To test for the presence of unidirectional driving by the $D_{2}$-based method we used time series $x_{1}$ and $y_{1}$ generated by Equation (1). First, we used C=0.48, which is well below the synchronization value. The starting point was [0.5,0.1,0.7,0.3]. The first 1000 data points were discarded and the next 100,000 were saved and used for $D_{2}$ estimations. We started with the reconstruction of $M_{X}$ and $M_{Y}$ using embedding parameters $m_{X}=2$, $\tau_{X}=1$, $m_{Y}=4$, and $\tau_{Y}=1$. Then we estimated $D_{2}$ for $M_{X}$ and got a value of about 1.22. The estimate of the correlation dimension for $M_{Y}$, as well as the estimate for $M_{\left[XY\right]}$ resulted in a value of about 2. Such an outcome correctly indicates that system *X* causes system *Y*. This test example is presented in the first row of Figure 2.

**Figure 3:**
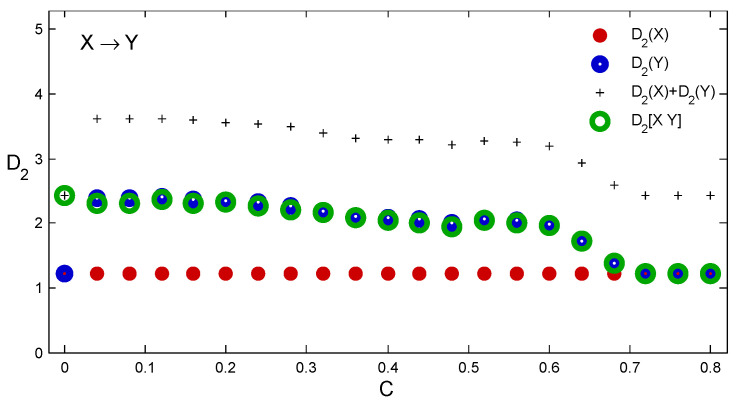
Estimates of $D_{2}\left(X\right)$ (red), $D_{2}\left(Y\right)$ (blue) and $D_{2}\left(\left[XY\right]\right)$ (green) of state portraits reconstructed from time series $x_{1}$ and $y_{1}$ generated by Equation (1) for 21 different values of coupling *C*. The plus signs are for the sums $D_{2}\left(X\right)+D_{2}\left(Y\right)$.

Recall, however, that we could identify the one-way causal link equally well for any other coupling value below the synchronization threshold. This is evident from Figure 3, where the $D_{2}$ estimates for increasing coupling strength *C* (see Equation (1)) are shown. $D_{2}\left(X\right)$ of about 1.22 was estimated for the driving system represented by $M_{X}$ and values of $D_{2}$ markedly higher for $M_{Y}$ and $M_{\left[XY\right]}$. Figure 3 also clearly reveals the onset of synchronization by a drop of $D_{2}\left(Y\right)$ to the level given by the driving system for the coupling of about 0.7. For the coupling value C=0 the result depends on the starting points of the two time series. Since we started here from different points, [0.5,0.1] and [0.7,0.3] respectively, we got two independent time series. As a result, $D_{2}\left(\left[XY\right]\right)=D_{2}\left(X\right)+D_{2}\left(Y\right)=2.44$. If the maps *X* and *Y* were started from the same point, we would get two identical time series and $D_{2}\left(X\right)=D_{2}\left(Y\right)=D_{2}\left(\left[XY\right]\right)=1.22$. The same applies for C>0.7, that is, after an identical synchronization, when the time series are no longer distinguishable.

Note also that it does not matter whether the data comes from maps or continuous dynamical systems. We used observables from Hénon maps here, but we could have equally well demonstrated the effectiveness of the method on data from flow systems. In [18] six different examples of linked Rössler and Lorenz systems have been presented, together with graphs of $D_{2}$ values for increasing coupling strength. The graphs have suggested that testing these examples would lead to equally clear results as those presented here.

### 3.2. *X* and *Y* Are Independent

As the second test example, we took observables $x_{1}$ and $y_{1}$ of Hénon maps (Equations (1) with C=0.48), generated with different starting points to get two independent time series. We determined $m_{X}=2$, $\tau_{X}=1$, $m_{Y}=4$, and $\tau_{Y}=1$ as the suitable embedding parameters for reconstructions of manifolds $M_{X}$ and $M_{Y}$. The correlation dimension of $M_{X}$ computed for 100,000 data points was found about $D_{2}\left(X\right)=1.22$. The estimate of $D_{2}\left(Y\right)$ from the more complex $M_{Y}$ was about 2.08. Then we concatenated $M_{X}$ and $M_{Y}$ to combine state vectors of both manifolds. The two dynamics were independent, each with its own degree of freedom, and for the dimension of the $M_{\left[XY\right]}$ we got an estimate of about 3.3, equal to the sum of the individual dimensions. This test case is shown in the second row of Figure 2.

### 3.3. Uncoupled *X* and *Y* with a Hidden Common Driver

**Figure 4:**
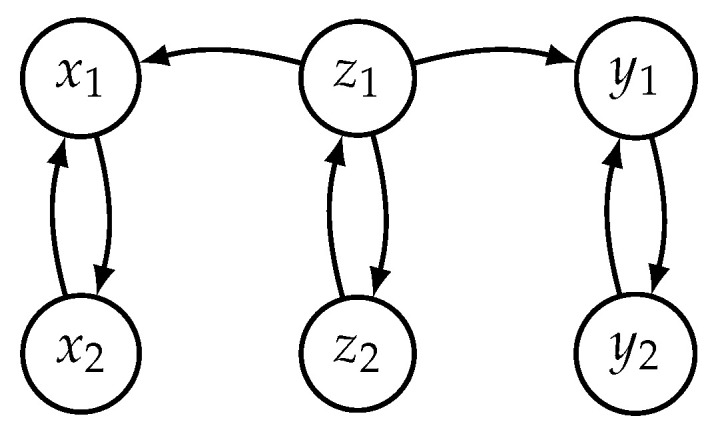
Interaction graph for two independent systems *X* and *Y* with a common driver *Z* described by Equation (2).

In the next example, we used two different Hénon maps to generate independent time series unidirectionally driven by a hidden common driver. The systems *X* and *Y* only differ in one parameter, which is set to 0.3 for the *X* case and 0.1 for the *Y* case. The first two lines correspond to the system *Z* driving both *X* and *Y*, while the systems *X* and *Y* are independent of each other (also see Figure 4 for the interaction graph):

(2) $$\begin{array}{cc} z_{1}\left(t+1\right) & =1.4-z_{1}^{2}\left(t\right)+0.3z_{2}\left(t\right)\\ z_{2}\left(t+1\right) & =z_{1}\left(t\right)\\ x_{1}\left(t+1\right) & =1.4-\left(Cz_{1}\left(t\right)x_{1}\left(t\right)+\left(1-C\right)x_{1}^{2}\left(t\right)\right)+0.3x_{2}\left(t\right)\\ x_{2}\left(t+1\right) & =x_{1}\left(t\right)\\ y_{1}\left(t+1\right) & =1.4-\left(Cz_{1}\left(t\right)y_{1}\left(t\right)+\left(1-C\right)y_{1}^{2}\left(t\right)\right)+0.1y_{2}\left(t\right)\\ y_{2}\left(t+1\right) & =y_{1}\left(t\right) \end{array}$$

Although, like in the second example, the systems are uncoupled, the signals $x_{1}$, $y_{1}$ might seem correlated or causally linked because they are controlled by a common hidden driver. We determined $m_{X}=4$, $\tau_{X}=1$, $m_{Y}=4$, and $\tau_{Y}=1$ as the suitable embedding parameters for reconstructions of $M_{X}$ and $M_{Y}$. Correlation dimensions computed for 100,000 data points were found to be about 2 and 2.1 for $D_{2}\left(X\right)$ and $D_{2}\left(Y\right)$, respectively. Then we combined the state vectors of both manifolds into 8-dimensional state space. Since the two dynamics were partly generated by a common driver, the estimated joint dimension of about 2.7 was, as expected, higher than the individual dimensions but less than the sum (4.1) of the two: $D_{2}\left(\left[XY\right]\right)<D_{2}\left(X\right)+D_{2}\left(Y\right)$. This example, for the hidden system driving with a strength of C=0.48, is presented in the third row of Figure 2.

**Figure 5:**
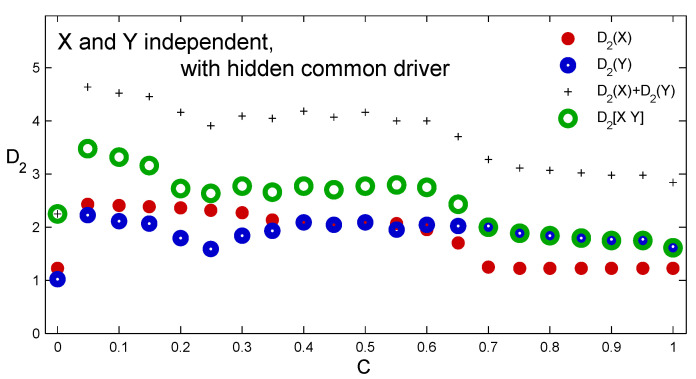
Estimates of $D_{2}\left(X\right)$ (red), $D_{2}\left(Y\right)$ (blue) and $D_{2}\left(\left[XY\right]\right)$ (green) of state portraits reconstructed from time series $x_{1}$ and $y_{1}$ generated by Equation (2) for different driving strength *C* of the hidden common driver. The plus signs are for the sums $D_{2}\left(X\right)+D_{2}\left(Y\right)$.

Figure 5, on the other hand, illustrates how sensitive the causality detection is to the driving strength of the hidden common driver. For this purpose, we generated and saved time series $x_{1}$, $y_{1}$, driven by *Z* using the coupling strength values *C* from 0 to 1 with the step of 0.05. In each case, the first 1000 data points were discarded and the next 100,000 were saved and used for $D_{2}$ estimations. For zero coupling (see Equation (1)) we have two unrelated systems of Hénon type: *X* with attractor of the complexity of about $D_{2}\left(X\right)=1.22$ and *Y* with attractor of the complexity of about $D_{2}\left(X\right)=1.02$. For increasing coupling strength the $D_{2}$ estimates indicate presence of a hidden common driver until *C* reaches the synchronization threshold at about 0.7. For higher couplings *X* is identically synchronized with the hidden driver *Z*, while *Y* remains driven but not synchronized [18]. *X* and *Z* become indistinguishable and the relations $D_{2}\left(\left[XY\right]\right)=D_{2}\left(Y\right)>D_{2}\left(X\right)$ suggest unidirectional link from *X* to *Y*.

### 3.4. X↔Y

As an example of bidirectional coupling, we used variables $y_{1}$ and $y_{2}$ generated by Equation (1), with C=0.48 and starting point of [0.5,0.1,0.7,0.3]. Let us denote $x=y_{1}$ and $y=y_{2}$. First of all, we made reconstructions of the state portraits. Both $M_{X}$ and $M_{Y}$ were reconstructed with embedding dimension m=4 and delay τ=1. Then the joined state space $M_{\left[XY\right]}$ was 8-dimensional. If *X* and *Y* interact bidirectionally, then, in theory, the cyclic flow of information ensures that any *x* or *y* variable contains information about the dynamics of both systems. Exactly in line with our expectations, all three estimates of dimensions reached the same value. See the shared plateau at about 2 in the last graph of the bottom row of Figure 2.

## 4. Discussion

In this study, we were facing an interesting problem of causality detection in cases, where the valid working hypothesis is that the investigated long time series *x* and *y* are manifestations of some dynamical systems *X* and *Y*, respectively.

If we analyzed autoregressive processes, we could use the Granger method as a tool for causal analysis. For dynamical systems, however, we must look for different approaches. Differences in causal analysis of dynamical systems as compared to autoregressive models have also emerged in connection with the first principle of Granger causality that the cause precedes the effect. Based on this principle one expects a change in the direction of causality from X→Y to Y→X when causally linked time series and their time-reversals are analyzed. It might even look like a good idea to use this turning test routinely to confirm the conclusion about the direction of the causal link. Indeed, this applies to autoregressive processes. However, Paluš et al. have pointed out that the expected change of the direction of causality did not happen after the time reversing of the tested chaotic signals [20]. The authors have suggested that the observed paradox is probably related to the dynamic memory of the systems. The lesson, among other things, is that in case of data from dynamical systems we should not try to confirm the direction of the causal link by analyzing the time-reversals.

Can correlation dimensions somehow contribute to this debate? $D_{2}$ as a geometrical characteristic is the same regardless of whether the points of the attractor are taken forward or backward in time. We only know that the dimension of the driving dynamical system (cause) is always lower than the dimension of the driven dynamical system (effect). Then, if the causal link is unidirectional, we can say that the direction of the coupling can only go from a system with a lower $D_{2}$ to the system with higher $D_{2}$.

To conclude, we must say that we consider the use of the correlation dimension in causal analysis to be a very promising approach, with a wide range of potential application areas. Many real data can be modeled by dynamical systems—usually through differential equations or discrete-time difference equations. The best-known examples include planetary motion, climate models, electric circuits, ecosystem and population dynamics modeling, cardio-respiratory interactions and other biomedical applications. The only requirement for the investigated time series is that they are generated by dynamics that can be modeled by dynamical systems and they are long and clean enough to enable estimating $D_{2}$ for the reconstructed systems.

However, many unanswered questions remain to be addressed. For example, the impact of noise needs to be examined. It is known that for noisy data the plateau for $D_{2}$ estimation is lifted and thus hardly evaluable. When applied to causality analysis, however, $D_{2}$ is interesting in terms of relative comparisons. In that case, if we expect noise to affect each time series evenly, meaningful results might still be obtained. We also need to be careful when the signals being investigated belong to the so-called 1/f processes. In such cases, $D_{2}$ estimates can easily be misinterpreted as a sign of low-dimensional dynamics [21]. It is one of the topics we would like to focus on soon because 1/f noise seems to be a ubiquitous feature of data from various areas including solids, condensed matter, electronic devices, music, economy, heart or brain signals.

Moving from pairwise causality detection to a multivariate approach is another issue that would be worth considering in future theoretical or computational studies.

What we know, for now, is that the $D_{2}$-based method offers unquestionable advantages in causal analysis of dynamical systems. If sufficiently long time series are available, then the detection of the causal relations is straightforward, fast, and reliable. It will be interesting to explore its possibilities and limitations in situations where deterministic dynamics do not play a dominant role in data. 

## Figures and Tables

**Table 1:** Rules for deriving causal relationships between systems *X* and *Y* based on dimensions $D_{2}\left(X\right)$, $D_{2}\left(Y\right)$ and $D_{2}\left(\left[XY\right]\right)$.

Causal Relation	Relations between Correlation Dimensions
X→Y	$D_{2}\left(\left[XY\right]\right)=D_{2}\left(Y\right)>D_{2}\left(X\right)$
Y→X	$D_{2}\left(\left[XY\right]\right)=D_{2}\left(X\right)>D_{2}\left(Y\right)$
*X* independent of *Y*	$D_{2}\left(\left[XY\right]\right)=D_{2}\left(X\right)+D_{2}\left(Y\right)$
*X* and *Y* uncoupled, with a common driver	$D_{2}\left(\left[XY\right]\right)<D_{2}\left(X\right)+D_{2}\left(Y\right)$, $D_{2}\left(X\right)<D_{2}\left(\left[XY\right]\right)>D_{2}\left(Y\right)$
X↔Y	$D_{2}\left(\left[XY\right]\right)=D_{2}\left(X\right)=D_{2}\left(Y\right)$
